# The Interplay between Autonomic Nervous System and Inflammation across Systemic Autoimmune Diseases

**DOI:** 10.3390/ijms23052449

**Published:** 2022-02-23

**Authors:** Chiara Bellocchi, Angelica Carandina, Beatrice Montinaro, Elena Targetti, Ludovico Furlan, Gabriel Dias Rodrigues, Eleonora Tobaldini, Nicola Montano

**Affiliations:** 1 Department of Internal Medicine, Fondazione IRCCS Ca’ Granda, Ospedale Maggiore Policlinico, 20122 Milan, Italy; angelica.carandina@unimi.it (A.C.); beatrice.montinaro@unimi.it (B.M.); elena.targetti@unimi.it (E.T.); ludovico.furlan@unimi.it (L.F.); eleonora.tobaldini@unimi.it (E.T.); 2 Department of Clinical Sciences and Community Health, University of Milan, 20122 Milan, Italy; gabriel.dias@unimi.it; 3 Laboratory of Experimental and Applied Exercise Physiology, Department of Physiology and Pharmacology, Fluminense Federal University, Niterói 24210-130, Brazil

**Keywords:** autonomic nervous system, sympathetic system, parasympathetic system, inflammatory reflex, innate immunity, adaptive immunity, systemic autoimmune diseases, inflammation, gut microbiota

## Abstract

The autonomic nervous system (ANS) and the immune system are deeply interrelated. The ANS regulates both innate and adaptive immunity through the sympathetic and parasympathetic branches, and an imbalance in this system can determine an altered inflammatory response as typically observed in chronic conditions such as systemic autoimmune diseases. Rheumatoid arthritis, systemic lupus erythematosus, and systemic sclerosis all show a dysfunction of the ANS that is mutually related to the increase in inflammation and cardiovascular risk. Moreover, an interaction between ANS and the gut microbiota has direct effects on inflammation homeostasis. Recently vagal stimulation techniques have emerged as an unprecedented possibility to reduce ANS dysfunction, especially in chronic diseases characterized by pain and a decreased quality of life as well as in chronic inflammation.

## 1. Introduction

The autonomic nervous system (ANS) has two main components, the sympathetic and the parasympathetic branches, that dynamically regulate the visceral functions [1]. Previous and recent findings are confirming the strong reciprocal interrelation between ANS and the immune system. Since ANS can regulate inflammation in chronic and acute conditions, autonomic dysfunction can have a pivotal influence on the onset and progression of many diseases where the immune response is involved, such as autoimmune diseases [2,3,4]. Given these premises, in the present review we will explore the interaction between immunity and ANS, focusing on the mutual contribution with both the innate and the adaptive immunity. Subsequently, we will explore from a more clinical point of view, what is currently known about ANS in three systemic autoimmune diseases in which immunity and inflammation are the main pathological processes such as rheumatoid arthritis (RA), systemic lupus erythematosus (SLE), and systemic sclerosis (SSc).

## 2. Autonomic Nervous System and Innate Immunity

The immune system is a complex interplay between immune cells, receptors, and self and non-self peptides. Innate immunity is the first response to microbes where pattern recognition receptors (PRRs) elicit the activation of immune/inflammatory processes after recognition of conserved pathogen-associated molecular patterns (PAMPs) that are present on bacteria, viruses, and fungi [5] or towards damage- (or danger-) associated molecular patterns (DAMPs) and others. Among PRRs, Toll-like receptors (TLRs), nod-like receptors, C-type lectin receptors, and many others have a role in inducing innate immune responses; when PRRs are present on antigen-presenting cells, mainly dendritic cells (DCs), they can induce also the adaptive immune response [6]. Furthermore, DCs play a pivotal role in contributing to either immune activation or maintaining the immune tolerance that is crucial to preventing autoimmunity [7,8]. Defensins, complement, granulocytes, and natural killer (NK) cells are components of innate immunity, determining inflammation that initially has a protective role from external or internal agents that have to be removed [5,9]. 

A deep interaction between the immune system and the nervous system is nowadays well documented and, in particular, innate immunity contributes to the development of the central nervous system (CNS) through microglia cells that are the main innate immune cells present in the brain [10,11]. TLRs are expressed on microglia surface responding to pathogenic or damaging insults [12,13,14]. Furthermore, immune cells and innate immune cells maintain the functioning and homeostasis of the nervous system and an imbalance of this equilibrium, due for example to chronic inflammation, can cause a severe impairment with consequent alteration of cognitive functions [15]. 

The interaction with innate immunity is not a prerogative of the CNS only and evidence show how the peripheral nervous system and more in specific the ANS have a deep interface with immune cells [16]. Anatomically, the sympathetic branch of ANS is present in immunological organs such as the thymus, spleen bone marrow, and lymph nodes while of interest, no evident traces of the parasympathetic fibers have been demonstrated [16,17,18,19,20,21,22]. Moreover, immune cells present adrenergic receptors able to bind norepinephrine that confers the ability to crosstalk with the sympathetic nerves, namely the alpha-adrenergic receptor (αAR) and the beta-adrenergic receptor (βAR), the latter more expressed on innate immune cells [23,24]. The receptors αAR and βAR have effects in opposite directions, where αAR can be considered more stimulatory, while βAR is inhibitory, and under homeostatic conditions, βAR has an overall predominant effect [25]. Altogether, from several studies, norepinephrine inhibits cytokines production, namely TNFα when expressed from monocytes, macrophage, and microglia in response to the lipopolysaccharide (LPS) constituent of the bacterial cell-wall as well as inhibits IL-1β or IL-6 production [26,27,28,29,30]. Norepinephrine has a direct effect on innate immune cells, increasing circulating NKs and granulocytes [31,32,33]. Neutrophil chemotaxis and phagocytosis are negatively regulated from norepinephrine; the NK function impairment after stroke seems to be mediated by a noradrenergic neurotransmitter, and the NK response is suppressed by catecholamines [34,35,36,37,38,39,40]. The βAR mediated effect of catecholamines suppress macrophage functions including their cytokine production [41,42]. Overall, the activation of the sympathetic nervous system attenuates the innate immunity as also demonstrated in a randomized control trial on human healthy subjects [43].

The parasympathetic branch that includes the vagus nerve has several effects on the innate immune system through the interaction between receptors present on the cellular surface and neurotransmitters, namely acetylcholine [44]. This communication is bidirectional and happens despite the fact that anatomically the parasympathetic fibers have not been individuated in the main immunologic organs such as the spleen and thymus [16,22]. Through vagal afferent fibers, the message that inflammation is present in other body sites reaches the CNS, as demonstrated by animal models of vagotomy in which the lack of vagal contribution determines reduced central responses with a blunted increase in body temperature and cortisol production [45,46,47]. Evidence of how the vagal afferents are activated by inflammation is not yet completely clear, but it has been suggested that IL-1β receptors, present especially in the vagal paraganglia, are the main promotors of this afferent reflex to the CNS; moreover, IL-1β is itself a key contributor in the direct stimulation of the brain to activate the inflammatory cascade [48,49].

The vagus nerve has an anti-inflammatory effect through the release of acetylcholine, mainly through the interaction with the α7 nicotinic acetylcholine receptor (α7nAChR) present on macrophages [50]. On cultures of LPS-stimulated human macrophages, acetylcholine attenuates the production of TNF, as well as of IL-6 and IL-1β, but not of the anti-inflammatory cytokine IL-10 [51]. The spleen is one of the main targets of vagal action towards the immune system. Indeed, it has been demonstrated how vagal stimulation reduces TNF macrophage production in mice sepsis models [52]. Due to the lack of parasympathetic fibers in the spleen, it has been hypothesized that the innervation is provided by catecholaminergic fibers from the celiac-superior mesenteric plexus ganglia that are under the control of preganglionic neurons of the thoracic spinal cord gray column [19,53,54,55]. Recently, an electrophysiological study performed on rats excluded the presence of a direct vagal-splenic nerve connection supporting the hypothesis of an effect towards splenic nerves mediated by vagal afferences through the CNS [21,33]. This neuronal modulation of inflammation through vagal afferences and efferences has been termed the “inflammatory reflex” [56]. Overall, the inflammatory reflex is crucial to maintain homeostasis with a balance between pro and anti-inflammatory responses as evident by the increase in morbidity and mortality during sepsis when a vagal depression is present [57,58,59,60]. 

## 3. Autonomic Nervous System and Adaptive Immunity

Adaptive immunity is the specialized branch of immunity able to respond to specific pathogens and to maintain an immunological memory over time. The main cells involved are lymphocytes B and T. The sympathetic nervous system is able to regulate the mobilization of lymphocytes in the bloodstream through catecholamines that directly interact with β2AR present on the lymphocytes’ surface [61]. Moreover, β2AR is selectively expressed on naïve T cells, CD4+ T helper (Th) 1, and regulatory T cells (Tregs) and induces T helper differentiation towards a Th1 phenotype through IFNγ/IL-12 interaction in in vitro studies, while in in vivo the Th differentiation is orchestrated via DCs+ [62,63]. Norepinephrine has an inhibitory effect on cytotoxic CD8+ T cells and modulates Tregs [64,65,66]. Regarding B cells, catecholamines have an indirect effect on their maturation and on antibodies production through their action on T cells that are necessary as costimulation in the B mediated immune responses [63]. Evidence on a direct effect of β2AR on B cells is limited; a lack of norepinephrine prevents a normal expression of IgG in mice [67] and norepinephrine induces β2AR mediated CD86 expression (a costimulator) on B cells [68,69].

Vagal stimulation increases acetylcholine release in the spleen and suppresses TNF-α in control BALB/c mice models of endotoxemia, while it does not reduce TNF-α in nude mice, suggesting that T cells are involved in the inflammatory reflex and that a T cell deficiency impairs the inflammatory reflex [70]. Moreover, α7nAChR present in T cells also causes a decrease in adhesion molecules expression and lymphocyte proliferation and both nicotinic and muscarinic acetylcholine receptors are present in lymphocytes that regulate their activities producing acetylcholine in a paracrine/autocrine control [71,72]. The role of vagal stimulation in increasing acetylcholine with beneficial effects on inflammation has been recently suggested also in the postural orthostatic tachycardia syndrome (POTS). POTS is a condition characterized by an impairment of the neuromodulation and consequent dysautonomia. Different studies showed a role of autoantibodies in POTS suggesting an autoimmune mediated pathogenesis of this condition [73,74]. In a recent study on a rabbit model of POTS induced by M2 muscarinic acetylcholine receptor-activating autoantibodies immunization, transcutaneous vagus nerve stimulation contributes to increasing acetylcholine with consequent reduction in both inflammation and cardiovagal dysfunction [75].

**Figure 1:**
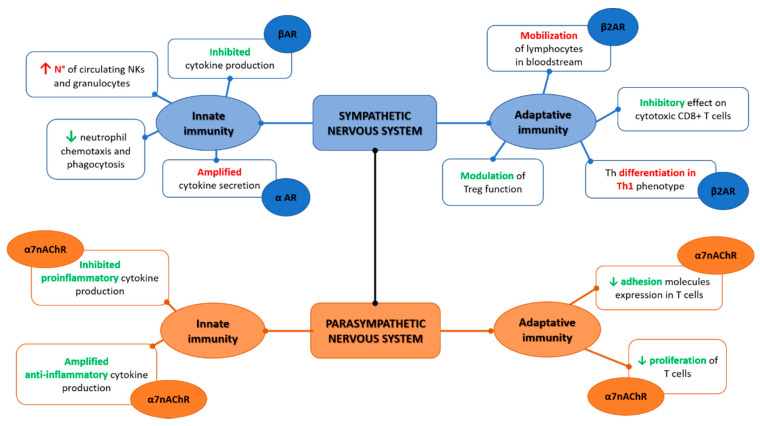
Sympathetic and parasympathetic synergic function on the innate and adaptive immunity.

Overall, once the inflammatory reflex is activated, the sympathetic and parasympathetic branches of ANS act synergistically instead of oppositely as intuitively expected Figure 1. Indeed, as elegantly depicted by Tracey [56], this synergic contribution implies that the vagal afferent fibers signal to the CNS (mainly within the nucleus of tractus solitarius) the presence of peripheric inflammatory/infective stimulus (intercepted for cytokines release and/or pathogens presence), and in response, vagal efferent fibers suppress cytokine release through nicotinic receptors present on macrophages, and throughout the cholinergic anti-inflammatory pathway. At the same time, the pain caused by the ongoing inflammatory processes can activate the sympathetic branches through the flight-or-fight responses determining norepinephrine release and consequent suppression of inflammation (via the pathways already detailed above) [76,77].

It is important to add that these mechanisms can have different implications and functioning in acute versus chronic conditions, as described in acute stress that can cause an immune hyperactivation, while chronic stress is typically associated with an immunosuppressive status [78], and what keeps the homeostasis is the dynamic balance between all these regulatory systems; when one system is prevailing, the imbalance can cause, or be the consequence of, a pathological condition, such as for example, chronic autoimmune diseases [79]. Finally, by way of example of the deep bidirectional complex interactions between the nervous system and both innate and adaptive immunity, we could considered the case of celiac disease (CD) in which evidence shows how both innate and adaptive immunity mechanisms are involved [80]. A wide range of neurological disorders, ANS dysfunction included, mediated by antineuronal and antigangliosides autoantibodies have been indeed demonstrated in CD [81,82,83,84].

## 4. Autonomic Nervous System and Gut Microbiota

The gastrointestinal tract (GIT) is considered one of the most extended and important immunological organs because of its enormous abundance of cells of both innate and adaptive immunity residing in the bowel mucosa [85]. In the GIT, the immune system directly interacts with the unique microbiota ecosystems that are hosted there; microbiota includes the whole composition of bacteria, fungi, and viruses that are present in a specific body site, and the gut microbiota has a crucial role from birth, allowing the evolution and development of the immune system as demonstrated by germ-free mice models in which the absence of microbiota is associated with an absent or impaired immune development [86,87,88,89]. Moreover, GIT microbiota can regulate the immune interaction with external antigens and maintain the immune homeostasis through its protolerogenic commensal Phyla of bacteria able to metabolize and generate short-chain fatty acids (butyrate, propionate, and acetate) that induce Tregs expansion in the colon [90,91]. A reduction in pro-tolerogenic bacteria, mainly Firmicutes and Bacteroides has been extensively described in studies performed on mice models and in patients with inflammatory bowel diseases (IBD) and irritable bowel syndrome (IBS) as well as in systemic autoimmune diseases [92,93,94,95]. 

The brain–gut axis (BGA) is a well-known interaction between the enteric nervous system (ENS) and CNS that also occurs through the sympathetic and parasympathetic branches of ANS [96,97]. Gut microbiota can directly interact with the ENS and indirectly modulate the BGA through neuroendocrine and neuroimmune pathways, all together considered the “brain–gut–microbiota” axis [98,99]. If these mechanisms undergo a dysfunction, an imbalance of this system leads to clinical alteration of the GIT especially with IBS development [100,101,102]. Moreover, the microbiota is directly associated with mental health disorders as demonstrated in knock-out mice models in which the absence of intestinal microbiota influences the development of behavior, along with neurochemical changes in the brain [102,103]. Microbiota alterations can modulate both the brain functions and the ANS through the vagus nerve, sending signals to the CNS and vice versa [104,105,106,107]. A recent study on beta 1 and 2 adrenergic receptor knock-out mice shows that the overall sympathetic reduction increases protolerogenic bacteria, with reduction in circulating CD4+ T cells and reduced IL-17 [108].

## 5. Autonomic Nervous System and Its Interplay with Inflammation in Systemic Autoimmune Diseases

### 5.1. Rheumatoid Arthritis

Rheumatoid arthritis (RA) is the most common form of chronic inflammatory arthritis, affecting 1% of the Western population [109]. It represents a real burden on public health as its prevalence is rising with high rates of disability and premature death mainly due to cardiovascular (CV) complications [110]. Patients with RA frequently experience physical disability due to persistent synovial inflammation that eventually leads to joint/bone deformities and chronic pain. Cardiovascular (e.g., ischemic heart disease, heart failure, arrhythmia) and psychiatric comorbidities are not infrequent in RA, and they can markedly impact patients’ quality of life [109]. 

As in other autoimmune diseases, it has been demonstrated that ANS plays a role in RA. Indeed, ANS dysfunction affects target organs (i.e., heart, kidney, and blood vessels), and it is also strongly interrelated with the onset and perpetuation of chronic inflammation via the ‘inflammatory reflex’ [111]. Signs of autonomic impairment in RA have been observed since the late 1950s [112]; however, the cause–effect and the temporal relationship between the onset of ANS dysfunction and inflammation is still a matter of current research. According to a systematic review based on 40 studies, the prevalence rate of ANS dysfunction among RA patients is 60%. Most of the studies report a reduction in cardiac parasympathetic activity (*n* = 20/26 studies, prevalence 77%) assessed through a reduced heart rate variability (HRV), that is a noninvasive method to investigate the status of cardiovascular autonomic control, and a high Resting Heart Rate (RHR) [113]. Under a physical effort, when compared to healthy subjects, RA patients have a reduced chronotropic response to exercise and slower heart rate recovery post-maximal exercise test [114]. Sympathetic hyperactivity has been documented in approximately half of the studies included in the aforementioned review (*n* = 16/30 studies, prevalence 53%), but the evidence is weaker since its measurement tools (e.g., clinical cardiovascular tests, neuropeptide Y, serum chromogranin, urinary or plasmatic catecholamines, pupillary light reflex, etc.) are less validated, and the results can be altered by concurrent conditions such as arterial hypertension [115]. Parasympathetic nervous system (PNS) hypoactivation and sympathetic nervous system (SNS) hyperactivity may exert their pathogenetic effect directly impacting the CV system and through an impairment of the cholinergic anti-inflammatory pathway. In RA, an imbalance in PNS-SNS stimulation may lead to a defective release and binding of acetylcholine to the α7nAChR on splenic macrophages and fibroblast-like synoviocytes in the joints with a consequent uncontrolled release of proinflammatory cytokines (e.g., TNF, IL-1, and IL-6) and self-sustaining chronic inflammation of target organs (e.g., joint and bone damage and accelerated atherosclerosis) [2,111]. 

This model is supported by experimental studies on animals that showed that treatment with α7nAChR pharmacological agonists (nicotine, AR-R17779, or GTS-21) reduces the clinical severity of arthritis as well as the expression of proinflammatory cytokines and atherosclerotic plaque in the aorta [2,116]. On the other hand, α7nAChR knockout mice had higher plasmatic levels of TNF, higher disease severity scores, and joint destruction compared to wildtype [2,117]. Recent data support the existence of a complex interlinkage between ANS dysfunction, chronic inflammation, and disease severity. For example, in RA patients elevated CRP seems to be independently associated with a significant depression of HRV and with QTc prolongation, thus increasing the risk of tachyarrhythmia, when compared to healthy controls or patients with low CRP levels [110,118]. Moreover, in a study conducted on 30 patients with RA, an augmented SNS activity was demonstrated by showing that heart rate and Muscle Sympathetic Nerve Activity (MSNA) were increased, and Cardiac Baroreflex Sensitivity (cBRS) was reduced in RA patients compared to non-RA patients, independently from arterial hypertension presence. In this study, pain was strongly correlated with MSNA (positive correlation) and cBRS (negative correlation), while heart rate had a positive independent association with high sensitive CRP (hs-CRP) and disease activity (DAS28-CRP) [115]. 

Some authors argue that ANS disturbances may precede the onset of inflammation rather than be its consequence, even though the causal issue is still a matter of debate. Interestingly, subjects ‘at risk’ for developing RA, which is defined by the positivity of rheumatoid factor (IgM-RF) or anti-citrullinated peptide autoantibodies (ACPA) along with arthralgia or a family history of RA, have a similar resting heart rate to RA patients, that is higher than healthy subjects. ANS impairment may anticipate or predict the onset of clinical disease, as RHR was significantly higher at baseline in those patients who subsequently developed arthritis [119]. Of note, the inflammatory reflex was impaired in subjects with a marked ANS dysfunction, as α7nAChR was significantly less expressed on peripheral blood monocytes of RA subjects [119]. CV complications account for 50% of premature deaths in RA and lead to a two-fold risk of sudden cardiac death, mainly due to Ischemic Heart Disease, Congestive Heart Failure, and arrhythmias [110]. 

The immune–autonomic link may also have a crucial role in determining the onset and severity of psychiatric comorbidities observed in patients with RA and other autoimmune diseases. It is well known that RA patients suffer more frequently from Major Depressive Disorder (MDD) and anxiety disorders than the general population [111]. Many mechanisms have been proposed to explain this association. First, peripheral proinflammatory cytokines can directly induce central nervous system (CNS) cells and the hypothalamic–pituitary–adrenal axis (HPA) to overproduce further cytokines and cortisol. Secondly, such cytokines can also alter the metabolism of neurotransmitters (e.g., dopamine, serotonin, and glutamate) causing reduced neuroplasticity. Moreover, anxiety appears to be associated with increased levels of IL-17 in RA [120]. Novel research points out that biological immunosuppresive drugs (e.g., anti-TNFα and anti-IL6 drugs) have a beneficial effect on anxious and depressive symptoms, and interesting perspectives come from studies on vagal nerve stimulation, which appears to ameliorate depression, RA symptoms, and chronic pain [2,121].

### 5.2. Systemic Lupus Erythematous

SLE is a systemic autoimmune disease characterized by chronic inflammation, multiple autoantibodies production, immune-complex deposition, and involvement of several organs (joints, skin, lungs, kidneys, and central and peripheral nervous systems) leading to variable clinical presentation and disease severity [122]. Overall, an ANS dysfunction in SLE has been extensively documented, with a prevalence of the sympathetic activity along with a decreased parasympathetic tone [3,123,124,125]. In particular, a reduced HRV and index of increased sympathetic modulation, is seen in SLE subjects as demonstrated in a study performed on 35 SLE patients where impaired HRV was also associated with the increase in inflammatory cytokines such as TNF and with disease activity [3]. Several other studies found a decreased HRV in SLE [126,127,128,129,130]. It is widely known that ANS dysfunction is associated with the development of cardiovascular diseases (CVDs), where the sympathetic activity is a mediator of both the onset and progression of CVDs while on the other hand, the parasympathetic control seems to have a protective role with reduced mortality in CVDs [131,132,133]. In SLE patients, CVDs are a high cause of mortality, especially related to atherosclerosis, and CVD risk is doubled with respect to the general population [134,135]. It could be speculated that this higher cardiovascular risk in SLE reflects the ANS dysfunction mutually linked to chronic inflammation as already postulated for RA. For example, in a mice model of SLE, the restoration of the vagal cholinergic anti-inflammatory pathway, with pharmacological compounds, reduces blood pressure along with inflammation [136,137]. The cardiac autonomic dysfunction may be related to QTc prolongation in SLE as investigated in a study on 91 SLE patients [138]. Overall these studies pave the way for wider future investigations on ANS’ mutual contribution with inflammation to disease onset and disease severity, as well as its impact on the cardiovascular system and quality of life in patients affected by SLE.

### 5.3. Systemic Sclerosis

Systemic sclerosis (SSc) is a rare systemic autoimmune disease characterized by microvascular impairment, production of several specific autoantibodies, and deposition of fibrosis in the dermal layer, thus contributing to the typical skin thickening (scleroderma) and to the involvement of several major organs such as lungs, heart, gastrointestinal (GI) tract, and kidneys [139]. An ANS dysfunction could explain part of the processes involved in SSc clinical manifestations. For example, a sympathetic overactivity determines prevalent vasoconstriction that is a leading process in the Raynaud phenomenon, the most common and earliest manifestation of SSc due to microvascular damage and related to cold temperatures and stress situations [140]. As described in [141] an HRV impairment is indeed found to be associated with microvascular damage assessed through the nailfold videocapillaroscopy of SSc patients and, interestingly, a study performed on twenty-seven SSc patients found a positive correlation between digital microvascular damage and parasympathetic modulation that promoted VEGF release to stimulate vasodilatation [142].

Along with sympathetic overactivity, ANS dysfunction is characterized also by a decreased parasympathetic function [143]. It begins in the early stages of SSc along with the organ fibrotic involvement (especially of the heart) and correlates with the disease subsets; the more fibrotic forms, such as diffuse cutaneous SSc, present a higher ANS impairment, while patients at the preclinical stage are similar to healthy subjects [144,145,146]. The presence of cardiac autonomic dysfunction has been extensively investigated in scleroderma patients and is associated with several cardiac manifestations, including left and right ventricular remodeling and cardiac repolarization abnormalities [147,148,149,150,151]. Moreover, HRV at rest is associated with the risk of developing arrhythmias as well as with mortality, and HRV response is impaired in SSc, when compared to healthy subjects during orthostatic stress [141,146,152]. Moreover, the role of ANS in mediating kidney vascular involvement has been studied [4]. The authors postulated that the increased stiffness and a further increase in vascular resistance were the results of sympathetic hyperactivity with a consequent increase in renal resistances detectable in SSc patients. Moreover, the GI affection that occurs in about 90% of SSc patients, has been related to an ANS impairment, especially of the vagal branch, that in physiologic conditions controls both the GI motility and the normal stress response. In SSc, ANS dysfunction is associated with esophageal dysmotility and patients with a severe GI disease have more symptoms of dysautonomia with consequent emotional distress [153,154,155,156]. HRV was also used to assess the relationship between dysautonomic symptoms and quality of life in SSc patients, showing that cardiovascular dysautonomia can be related to poor sleep quality, high pain scores, and depressive symptoms with an overall severe impact on quality of life [157].

## 6. Present and Future Perspectives

Based on the premises above, a substantial amount of data is emerging regarding the effects of vagal stimulation on the neuroinflammatory regulation, not only in models of endotoxemia but also in preclinical models of autoimmune diseases (specifically RA and inflammatory bowel diseases (IBD)) in which vagal stimulation can control inflammatory/immune activation [52,158,159,160,161]. New clinical trials that implicate the use of neurostimulators for RA and IBD are emerging [162,163,164,165]. Vagal stimulation is nowadays investigated as an efficacious tool to obtain relief from chronic pain and depression pain-related symptoms [166,167,168]. Furthermore, a direct noninvasive modulation of the vagal nerve using transcutaneous vagus nerve stimulation (tVNS) represents a promising non-pharmacological and noninvasive therapy for cardiovascular and noncardiovascular disorders [169,170].

In RA, VNS might have a beneficial anti-inflammatory effect in patients who did not fully respond to drug therapy (corticosteroids and synthetic or biologic disease-modifying antirheumatic drugs). Indeed, in a study after 42 days of 60 s stimulation 1–4 times daily, there was a drop in TNF levels and a significant clinical improvement of at least 20% in approximately 70% of patients treated. These beneficial effects were nullified by a 2 week discontinuation of VNS, which was associated with an additional increment in TNF production and DAS28-CRP [2].

In SLE, the scientific background that justifies the future use of VNS is depicted in [171]. Moreover, recently a double-blind sham-controlled pilot study was conducted on 18 SLE patients with pain, in which tVNS significantly reduced pain and fatigue, compared to sham-stimulation and joint scores after 5 and 12 days [172]. One study investigated the effects of a tVNS on 17 SSc patients with upper GI tract dysfunction versus nine healthy controls, showing an altered HRV in SSc and normalization of sympatho-vagal balance with improvement of the GI symptoms score after prolonged use of tVNS [173].

**Figure 2:**
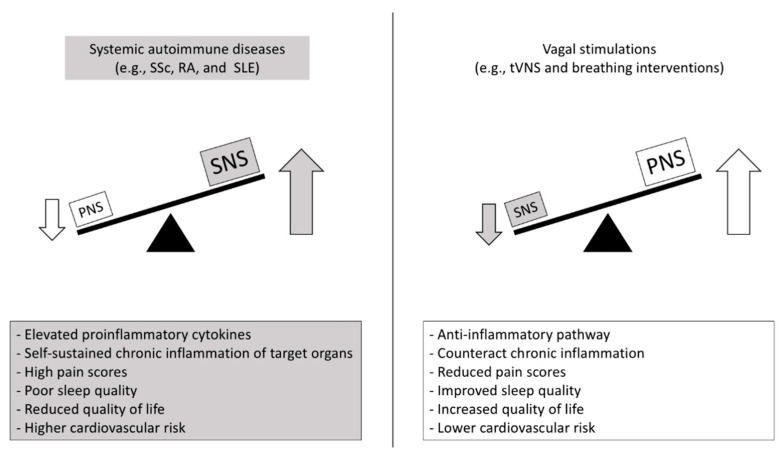
Putative autonomic- and inflammatory-mediated mechanisms and clinical outcomes in autoimmune diseases and promising countermeasures. SNS: sympathetic nervous system; PNS: parasympathetic nervous system; SSc: Systemic sclerosis; RA: Rheumatoid arthritis; SLE: Systemic lupus erythematous; tVNS: transcutaneous vagus nerve stimulation.

Therefore, advancing the knowledge of the interplay between the autonomic nervous system, inflammation, and autoimmune diseases could be a great opportunity for several fields, such as bioelectronics medicine. Since new pathophysiological mechanisms have been revealed, instruments and protocols could be developed, expanding the possibilities of nonpharmacological treatment and rehabilitation in autoimmune and inflammatory diseases. Figure 2.

## 7. Conclusions

The evidence of the interplay between ANS and the immune system are multifaceted and are at the basis of the clinical pictures (such as organ inflammation, pain, CV involvement, and fatigue) of diseases where chronic inflammation is implied. In particular, the potential benefit for a nonpharmacological intervention in systemic autoimmune diseases based on vagal stimulation is an emerging field of interest that is worth further study to confirm its efficacy in improving the symptoms and quality of life of these patients.

## Data Availability

Not applicable.

## References

[1] Sanvictores T., Tadi P. (2022). Neuroanatomy, Autonomic Nervous System Visceral Afferent Fibers and Pain.

[2] Koopman F.A., Van Maanen M.A., Vervoordeldonk M.J., Tak P.P. (2017). Balancing the autonomic nervous system to reduce inflammation in rheumatoid arthritis. J. Intern. Med..

[3] Thanou A., Stavrakis S., Dyer J.W., Munroe M.E., James J.A., Merrill J.T. (2016). Impact of heart rate variability, a marker for cardiac health, on lupus disease activity. Arthritis Res. Ther..

[4] Gigante A., Rosato E., Liberatori M., Barbano B., Cianci R., Gasperini M., Sardo L., Marra A., Amoroso A., Salsano F. (2014). Autonomic dysfunction in patients with systemic sclerosis: Correlation with intrarenal arterial stiffness. Int. J. Cardiol..

[5] Takeuchi O., Akira S. (2010). Pattern Recognition Receptors and Inflammation. Cell.

[6] Iwasaki A., Medzhitov R. (2015). Control of adaptive immunity by the innate immune system. Nat. Immunol..

[7] Shortman K., Caux C. (1997). Dendritic Cell Development: Multiple Pathways to Nature’s Adjuvants. Stem Cells.

[8] Banchereau J., Steinman R.M. (1998). Dendritic cells and the control of immunity. Nature.

[9] Nathan C. (2002). Points of control in inflammation. Nature.

[10] Korin B., Ben-Shaanan T.L., Schiller M., Dubovik T., Azulay-Debby H., Boshnak N.T., Koren T., Rolls A. (2017). High-dimensional, single-cell characterization of the brain’s immune compartment. Nat. Neurosci..

[11] Matcovitch-Natan O., Winter D.R., Giladi A., Aguilar S.V., Spinrad A., Sarrazin S., Ben-Yehuda H., David E., González F.Z., Perrin P. (2016). Microglia development follows a stepwise program to regulate brain homeostasis. Science.

[12] Olson J.K., Miller S.D. (2004). Microglia Initiate Central Nervous System Innate and Adaptive Immune Responses through Multiple TLRs. J. Immunol..

[13] Tang S.-C., Arumugam T.V., Xu X., Cheng A., Mughal M.R., Jo D.-G., Lathia J.D., Siler D.A., Chigurupati S., Ouyang X. (2007). Pivotal role for neuronal Toll-like receptors in ischemic brain injury and functional deficits. Proc. Natl. Acad. Sci. USA.

[14] Klein M., Obermaier B., Angele B., Pfister H., Wagner H., Koedel U., Kirschning C.J. (2008). Innate Immunity to Pneumococcal Infection of the Central Nervous System Depends on Toll-Like Receptor (TLR) 2 and TLR4. J. Infect. Dis..

[15] Zengeler K.E., Lukens J.R. (2021). Innate immunity at the crossroads of healthy brain maturation and neurodevelopmental disorders. Nat. Rev. Immunol..

[16] Nance D.M., Sanders V.M. (2007). Autonomic innervation and regulation of the immune system (1987–2007). Brain, Behav. Immun..

[17] Romeo H.E., Fink T., Yanaihara N., Weihe E. (1994). Distribution and relative proportions of neuropeptide Y- and proenkephalin-containing noradrenergic neurones in rat superior cervical ganglion: Separate projections to submaxillary lymph nodes. Peptides.

[18] Trotter R.N., Stornetta R.L., Guyenet P.G., Roberts M.R. (2007). Transneuronal mapping of the CNS network controlling sympathetic outflow to the rat thymus. Auton. Neurosci..

[19] Cano G., Sved A.F., Rinaman L., Rabin B.S., Card J.P. (2001). Characterization of the central nervous system innervation of the rat spleen using viral transneuronal tracing. J. Comp. Neurol..

[20] Bulay O., Mlrvish S.S., Pelfrene A.F., Eagen M., Garcia H., Gold B. (1979). Carcinogenicity Test of Six Nitrosamides and a Nitrosocyanamide Administered Orally to Rats2. JNCI: J. Natl. Cancer Inst..

[21] Bratton B.O., Martelli D., McKinley M.J., Trevaks D., Anderson C.R., McAllen R.M. (2012). Neural regulation of inflammation: No neural connection from the vagus to splenic sympathetic neurons. Exp. Physiol..

[22] Bellinger D., Lorton D., Hamill R., Felten S., Felten D. (1993). Acetylcholinesterase Staining and Choline Acetyltransferase Activity in the Young Adult Rat Spleen: Lack of Evidence for Cholinergic Innervation. Brain, Behav. Immun..

[23] Sanders V.M., Straub R.H. (2002). Norepinephrine, the β-Adrenergic Receptor, and Immunity. Brain, Behav. Immun..

[24] Sanders V.M., E Munson A. (1985). Norepinephrine and the antibody response. Pharmacol. Rev..

[25] Daaka Y., Luttrell L., Lefkowitz R.J. (1997). Switching of the coupling of the β2-adrenergic receptor to different G proteins by protein kinase A. Nature.

[26] Meltzer J.C., MacNeil B.J., Sanders V., Pylypas S., Jansen A.H., Greenberg A.H., Nance D.M. (2004). Stress-induced suppression of in vivo splenic cytokine production in the rat by neural and hormonal mechanisms. Brain, Behav. Immun..

[27] Ignatowski T., Gallant S., Spengler R.N. (1996). Temporal regulation by adrenergic receptor stimulation of macrophage (MΦ)-derived tumor necrosis factor (TNF) production post-LPS challenge. J. Neuroimmunol..

[28] Hetier E., Ayala J., Bousseau A., Prochiantz A. (1991). Modulation of interleukin-1 and tumor necrosis factor expression by ?-adrenergic agonists in mouse ameboid microglial cells. Exp. Brain Res..

[29] van der Poll T., Jansen J., Endert E., Sauerwein H.P., van Deventer S.J. (1994). Noradrenaline inhibits lipopolysaccharide-induced tumor necrosis factor and interleukin 6 production in human whole blood. Infect. Immun..

[30] Severn A., Rapson N.T., A Hunter C., Liew F.Y. (1992). Regulation of tumor necrosis factor production by adrenaline and beta-adrenergic agonists. J. Immunol..

[31] A Ottaway C. (1992). Central nervous system influences on lymphocyte migration. Brain, Behav. Immun..

[32] Benschop R.J., Rodriguez-Feuerhahn M., Schedlowski M. (1996). Catecholamine-Induced Leukocytosis: Early Observations, Current Research, and Future Directions. Brain Behav. Immun..

[33] Bellinger D.L., Lorton D. (2014). Autonomic regulation of cellular immune function. Auton. Neurosci..

[34] Nicholls A.J., Wen S.W., Hall P., Hickey M., Wong C.H.Y. (2017). Activation of the sympathetic nervous system modulates neutrophil function. J. Leukoc. Biol..

[35] Harvath L., Robbins J.D., A Russell A., Seamon K.B. (1991). cAMP and human neutrophil chemotaxis. Elevation of cAMP differentially affects chemotactic responsiveness. J. Immunol..

[36] Zurier R.B., Weissmann G., Hoffstein S., Kammerman S., Tai H.H. (1974). Mechanisms of Lysosomal Enzyme Release from Human Leukocytes II. EFFECTS OF cAMP AND cGMP, AUTONOMIC AGONISTS, AND AGENTS WHICH AFFECT MICROTUBULE FUNCTION. J. Clin. Investig..

[37] Wong C.H.Y., Jenne C.N., Lee W.-Y., Léger C., Kubes P. (2011). Functional Innervation of Hepatic iNKT Cells Is Immunosuppressive Following Stroke. Science.

[38] Irwin M. (1994). Stress-induced immune suppression: Role of brain corticotropin releasing hormone and autonomic nervous system mechanisms. Adv. Neuroimmunol..

[39] Elenkov I.J., Wilder R.L., Chrousos G.P., Vizi E.S. (2000). The sympathetic nerve--an integrative interface between two supersystems: The brain and the immune system. Pharmacol. Rev..

[40] Shakhar G., Ben-Eliyahu S. (1998). In vivo beta-adrenergic stimulation suppresses natural killer activity and compromises resistance to tumor metastasis in rats. J. Immunol..

[41] Suberville S., Bellocq A., Fouqueray B., Philippe C., Lantz O., Perez J., Baud L. (1996). Regulation of interleukin-10 production by β-adrenergic agonists. Eur. J. Immunol..

[42] Németh Z.H., Szabó C., Haskó G., Salzman A.L., Vizi E. (1997). Effect of the phosphodiesterase III inhibitor amrinone on cytokine and nitric oxide production in immunostimulated J774.1 macrophages. Eur. J. Pharmacol..

[43] Kox M., van Eijk L.T., Zwaag J., Wildenberg J.V.D., Sweep F., van der Hoeven J.G., Pickkers P. (2014). Voluntary activation of the sympathetic nervous system and attenuation of the innate immune response in humans. Proc. Natl. Acad. Sci. USA.

[44] Kox M., Pickkers P. (2015). Modulation of the Innate Immune Response through the Vagus Nerve. Nephron Exp. Nephrol..

[45] Gaykema R.P., Dijkstra I., Tilders F.J. (1995). Subdiaphragmatic vagotomy suppresses endotoxin-induced activation of hypothalamic corticotropin-releasing hormone neurons and ACTH secretion. Endocrinology.

[46] Fleshner M., Goehler L., Schwartz B., McGorry M., Martin D., Maier S., Watkins L. (1998). Thermogenic and corticosterone responses to intravenous cytokines (IL-1β and TNF-α) are attenuated by subdiaphragmatic vagotomy. J. Neuroimmunol..

[47] Huston J.M., Ochani M., Rosas-Ballina M., Liao H., Ochani K., Pavlov V., Puerta M., Ashok M., Czura C.J., Foxwell B. (2006). Splenectomy inactivates the cholinergic antiinflammatory pathway during lethal endotoxemia and polymicrobial sepsis. J. Exp. Med..

[48] Goehler L.E., Relton J.K., Dripps D., Kiechle R., Tartaglia N., Maier S.F., Watkins L.R. (1997). Vagal Paraganglia Bind Biotinylated Interleukin-1 Receptor Antagonist: A Possible Mechanism for Immune-to-Brain Communication. Brain Res. Bull..

[49] van Westerloo D.J. (2010). The vagal immune reflex: A blessing from above. Wien. Med. Wochenschr..

[50] Wang H., Yu M., Ochani M., Amella C.A., Tanovic M., Susarla S., Li J.H., Wang H., Yang H., Ulloa L. (2003). Nicotinic acetylcholine receptor α7 subunit is an essential regulator of inflammation. Nature.

[51] Borovikova L.V., Ivanova S., Zhang M., Yang H., Botchkina G.I., Watkins L.R., Wang H., Abumrad N., Eaton J.W., Tracey K.J. (2000). Vagus nerve stimulation attenuates the systemic inflammatory response to endotoxin. Nature.

[52] Rosas-Ballina M., Ochani M., Parrish W.R., Ochani K., Harris Y.T., Huston J.M., Chavan S., Tracey K.J. (2008). Splenic nerve is required for cholinergic antiinflammatory pathway control of TNF in endotoxemia. Proc. Natl. Acad. Sci. USA.

[53] Nance D.M., Burns J. (1989). Innervation of the spleen in the rat: Evidence for absence of afferent innervation. Brain Behav. Immun..

[54] Hosoi T., Okuma Y., Matsuda T., Nomura Y. (2005). Novel pathway for LPS-induced afferent vagus nerve activation: Possible role of nodose ganglion. Auton. Neurosci..

[55] Vida G., Peña G., Deitch E.A., Ulloa L. (2011). α7-Cholinergic Receptor Mediates Vagal Induction of Splenic Norepinephrine. J. Immunol..

[56] Tracey K.J. (2002). The inflammatory reflex. Nature.

[57] Pontet J., Contreras P., Curbelo A., Medina J., Noveri S., Bentancourt S., Migliaro E.R. (2003). Heart rate variability as early marker of multiple organ dysfunction syndrome in septic patients. J. Crit. Care.

[58] Pavlov V.A., Ochani M., Yang L.-H., Gallowitsch-Puerta M., Ochani K., Lin X., Levi J., Parrish W.R., Rosas-Ballina M., Czura C.J. (2007). Selective α7-nicotinic acetylcholine receptor agonist GTS-21 improves survival in murine endotoxemia and severe sepsis*. Crit. Care Med..

[59] Koh D.-R., Fung-Leung W.-P., Ho A., Gray D., Acha-Orbea H., Mak T.-W. (1992). Less Mortality but More Relapses in Experimental Allergic Encephalomyelitis in CD8 -/- Mice. Science.

[60] Bernik T.R., Friedman S.G., Ochani M., DiRaimo R., Susarla S., Czura C.J., Tracey K.J. (2002). Cholinergic antiinflammatory pathway inhibition of tumor necrosis factor during ischemia reperfusion. J. Vasc. Surg..

[61] Dimitrov S., Lange T., Born J. (2009). Selective Mobilization of Cytotoxic Leukocytes by Epinephrine. J. Immunol..

[62] Guereschi M.G., Araujo L.P., Maricato J.T., Takenaka M.C., Nascimento V.M., Vivanco B.C., Reis V.O., Keller A.C., Brum P.C., Basso A.S. (2013). Beta2-adrenergic receptor signaling in CD4+Foxp3+regulatory T cells enhances their suppressive function in a PKA-dependent manner. Eur. J. Immunol..

[63] Sanders V.M. (2012). The beta2-adrenergic receptor on T and B lymphocytes: Do we understand it yet?. Brain, Behav. Immun..

[64] Wirth T., Westendorf A.M., Bloemker D., Wildmann J., Engler H., Mollerus S., Wadwa M., Schäfer M.K.-H., Schedlowski M., del Rey A. (2014). The sympathetic nervous system modulates CD4+Foxp3+ regulatory T cells via noradrenaline-dependent apoptosis in a murine model of lymphoproliferative disease. Brain, Behav. Immun..

[65] Kalinichenko V.V., Mokyr M.B., Graf L.H., Cohen R.L., A Chambers D. (1999). Norepinephrine-mediated inhibition of antitumor cytotoxic T lymphocyte generation involves a beta-adrenergic receptor mechanism and decreased TNF-alpha gene expression. J. Immunol..

[66] Livnat S., Madden K.S., Felten D.L., Felten S.Y. (1987). Regulation of the immune system by sympathetic neural mechanisms. Prog. Neuro-Psychopharmacol. Biol. Psychiatry.

[67] Kohm A.P., Sanders V.M. (1999). Suppression of antigen-specific Th2 cell-dependent IgM and IgG1 production following norepinephrine depletion in vivo. J. Immunol..

[68] Kohm A.P., Mozaffarian A., Sanders V.M. (2002). B Cell Receptor- and β2-Adrenergic Receptor-Induced Regulation of B7-2 (CD86) Expression in B Cells. J. Immunol..

[69] Kasprowicz D.J., Kohm A.P., Berton M.T., Chruscinski A.J., Sharpe A.H., Sanders V.M. (2000). Stimulation of the B Cell Receptor, CD86 (B7-2), and the β2-Adrenergic Receptor Intrinsically Modulates the Level of IgG1 and IgE Produced per B Cell. J. Immunol..

[70] Rosas-Ballina M., Olofsson P.S., Ochani M., Valdés-Ferrer S.I., Levine Y.A., Reardon C., Tusche M.W., Pavlov V.A., Andersson U., Chavan S. (2011). Acetylcholine-Synthesizing T Cells Relay Neural Signals in a Vagus Nerve Circuit. Science.

[71] Geng Y., Savage S., Johnson L., Seagrave J., Sopori M. (1995). Effects of Nicotine on the Immune Response. I. Chronic Exposure to Nicotine Impairs Antigen Receptor-Mediated Signal Transduction in Lymphocytes. Toxicol. Appl. Pharmacol..

[72] Kawashima K. (2000). Extraneuronal cholinergic system in lymphocytes. Pharmacol. Ther..

[73] Vernino S., Stiles L.E. (2018). Autoimmunity in postural orthostatic tachycardia syndrome: Current understanding. Auton. Neurosci..

[74] Li H., Yu X., Liles C., Khan M., Vanderlinde-Wood M., Galloway A., Zillner C., Benbrook A., Reim S., Collier D. (2014). Autoimmune Basis for Postural Tachycardia Syndrome. J. Am. Hear. Assoc..

[75] Deng J., Li H., Guo Y., Zhang G., Fischer H., Stavrakis S., Yu X. (2022). Transcutaneous vagus nerve stimulation attenuates autoantibody-mediated cardiovagal dysfunction and inflammation in a rabbit model of postural tachycardia syndrome. J. Interv. Card. Electrophysiol..

[76] Molina P.E. (2001). Noradrenergic inhibition of TNF upregulation in hemorrhagic shock. Neuroimmunomodulation.

[77] Woiciechowsky C., Asadullah K., Nestler D., Eberhardt B., Platzer C., Schöning B., Glöckner F., Lanksch W.R., Volk H.-D., Döcke W.-D. (1998). Sympathetic activation triggers systemic interleukin-10 release in immunodepression induced by brain injury. Nat. Med..

[78] Dhabhar F.S. (2008). Enhancing versus Suppressive Effects of Stress on Immune Function: Implications for Immunoprotection versus Immunopathology. Allergy, Asthma Clin. Immunol..

[79] Pongratz G., Straub R.H. (2014). The sympathetic nervous response in inflammation. Arthritis Res. Ther..

[80] Voisine J., Abadie V. (2021). Interplay between Gluten, HLA, Innate and Adaptive Immunity Orchestrates the Development of Coeliac Disease. Front. Immunol..

[81] Cervio E., Volta U., Verri M., Boschi F., Pastoris O., Granito A., Barbara G., Parisi C., Felicani C., Tonini M. (2007). Sera of Patients With Celiac Disease and Neurologic Disorders Evoke a Mitochondrial-Dependent Apoptosis In Vitro. Gastroenterology.

[82] Volta U., De Giorgio R., Granito A., Stanghellini V., Barbara G., Avoni P., Liguori R., Petrolini N., Fiorini E., Montagna P. (2006). Anti-ganglioside antibodies in coeliac disease with neurological disorders. Dig. Liver Dis..

[83] Kayali S., Selbuz S. (2020). Assessment of Autonomic Nervous System in Children with Celiac Disease: A Heart Rate Variability Study. Indian Pediatr..

[84] Przybylska-Felus M., Furgala A., Zwolinska-Wcislo M., Mazur M., Widera A., Thor P., Mach T. (2014). Disturbances of autonomic nervous system activity and diminished response to stress in patients with celiac disease. J. Physiol. Pharmacol. Off. J. Pol. Physiol. Soc..

[85] Guy-Grand D., DiSanto J.P., Henchoz P., Malassis-Séris M., Vassalli P. (1998). Small bowel enteropathy: Role of intraepithelial lymphocytes and of cytokines (IL-12, IFN-gamma, TNF) in the induction of epithelial cell death and renewal. Eur. J. Immunol..

[86] Sekirov I., Russell S.L., Antunes L.C.M., Finlay B.B. (2010). Gut Microbiota in Health and Disease. Physiol. Rev..

[87] Sprockett D., Fukami T., Relman D.A. (2018). Role of priority effects in the early-life assembly of the gut microbiota. Nat. Rev. Gastroenterol. Hepatol..

[88] Palmer C., Bik E.M., DiGiulio D.B., Relman D.A., Brown P.O. (2007). Development of the Human Infant Intestinal Microbiota. PLoS Biol..

[89] Umesaki Y., Setoyama H., Matsumoto S., Okada Y. (1993). Expansion of alpha beta T-cell receptor-bearing intestinal intraepithelial lymphocytes after microbial colonization in germ-free mice and its independence from thymus. Immunology.

[90] Macfarlane S., Macfarlane G.T. (2003). Regulation of short-chain fatty acid production. Proc. Nutr. Soc..

[91] Bellocchi C., Volkmann E.R. (2018). Update on the Gastrointestinal Microbiome in Systemic Sclerosis. Curr. Rheumatol. Rep..

[92] Seksik P., Rigottier-Gois L., Gramet G., Sutren M., Pochart P., Marteau P., Jian R., Doré J. (2003). Alterations of the dominant faecal bacterial groups in patients with Crohn’s disease of the colon. Gut.

[93] Pozuelo M., Panda S., Santiago A., Mendez S., Accarino A., Santos J., Guarner F., Azpiroz F., Manichanh C. (2015). Reduction of butyrate- and methane-producing microorganisms in patients with Irritable Bowel Syndrome. Sci. Rep..

[94] Bellocchi C., Fernández-Ochoa Á., Montanelli G., Vigone B., Santaniello A., Milani C., Quirantes-Piné R., Borrás-Linares I., Ventura M., Segura-Carrettero A. (2018). Microbial and metabolic multi-omic correlations in systemic sclerosis patients. Ann. N. Y. Acad. Sci..

[95] Chen B., Jia X., Xu J., Zhao L., Ji J., Wu B., Ma Y., Li H., Zuo X., Pan W. (2020). An Autoimmunogenic and Proinflammatory Profile Defined by the Gut Microbiota of Patients With Untreated Systemic Lupus Erythematosus. Arthritis Rheumatol..

[96] Tait C., Sayuk G.S. (2021). The Brain-Gut-Microbiotal Axis: A framework for understanding functional GI illness and their therapeutic interventions. Eur. J. Intern. Med..

[97] Mayer E.A. (2011). Gut feelings: The emerging biology of gut–brain communication. Nat. Rev. Neurosci..

[98] Mayer E.A., Knight R., Mazmanian S.K., Cryan J.F., Tillisch K. (2014). Gut Microbes and the Brain: Paradigm Shift in Neuroscience. J. Neurosci..

[99] Martin C.R., Osadchiy V., Kalani A., Mayer E.A. (2018). The Brain-Gut-Microbiome Axis. Cell. Mol. Gastroenterol. Hepatol..

[100] Mayer E.A., Tillisch K. (2011). The Brain-Gut Axis in Abdominal Pain Syndromes. Annu. Rev. Med..

[101] Berman S.M., Naliboff B.D., Suyenobu B., Labus J.S., Stains J., Ohning G., Kilpatrick L., Bueller J.A., Ruby K., Jarcho J. (2008). Reduced Brainstem Inhibition during Anticipated Pelvic Visceral Pain Correlates with Enhanced Brain Response to the Visceral Stimulus in Women with Irritable Bowel Syndrome. J. Neurosci..

[102] Neufeld K.M., Kang N., Bienenstock J., Foster J.A. (2010). Reduced anxiety-like behavior and central neurochemical change in germ-free mice. Neurogastroenterol. Motil..

[103] Kelly J., Borre Y., Brien C.O., Patterson E., El Aidy S., Deane J., Kennedy P.J., Beers S., Scott K., Moloney G. (2016). Transferring the blues: Depression-associated gut microbiota induces neurobehavioural changes in the rat. J. Psychiatr. Res..

[104] Bonaz B., Sinniger V., Pellissier S. (2017). The Vagus Nerve in the Neuro-Immune Axis: Implications in the Pathology of the Gastrointestinal Tract. Front. Immunol..

[105] Furness J.B. (2016). Integrated Neural and Endocrine Control of Gastrointestinal Function. Enteric Nerv. Syst..

[106] Diepenbroek C., Quinn D., Stephens R., Zollinger B., Anderson S., Pan A., De Lartigue G. (2017). Validation and characterization of a novel method for selective vagal deafferentation of the gut. Am. J. Physiol. Liver Physiol..

[107] Powell N., Walker M.M., Talley N.J. (2017). The mucosal immune system: Master regulator of bidirectional gut–brain communications. Nat. Rev. Gastroenterol. Hepatol..

[108] Bartley A., Yang T., Arocha R., Malphurs W.L., Larkin R., Magee K.L., Vickroy T.W., Zubcevic J. (2018). Increased Abundance of Lactobacillales in the Colon of Beta-Adrenergic Receptor Knock Out Mouse Is Associated With Increased Gut Bacterial Production of Short Chain Fatty Acids and Reduced IL17 Expression in Circulating CD4+ Immune Cells. Front. Physiol..

[109] Scott D.L., Wolfe F., Huizinga T.W.J. (2010). Rheumatoid arthritis. Lancet.

[110] Lazzerini P.E., Capecchi P.L., Acampa M., Galeazzi M., Pasini F.L. (2014). Arrhythmic risk in rheumatoid arthritis: The driving role of systemic inflammation. Autoimmun. Rev..

[111] Ingegnoli F., Buoli M., Antonucci F., Coletto L.A., Esposito C.M., Caporali R. (2020). The Link between Autonomic Nervous System and Rheumatoid Arthritis: From Bench to Bedside. Front. Med..

[112] Hart F.D., Golding J.R., Mackenzie D.H. (1957). Neuropathy in Rheumatoid Disease. Ann. Rheum. Dis..

[113] Adlan A.M., Lip G.Y., Paton J.F., Kitas G., Fisher J. (2014). Autonomic function and rheumatoid arthritis—A systematic review. Semin. Arthritis Rheum..

[114] Peçanha T., Rodrigues R., Pinto A.J., Guedes L., Bonfiglioli K., Gualano B., Roschel H. (2018). Chronotropic Incompetence and Reduced Heart Rate Recovery in Rheumatoid Arthritis. JCR: J. Clin. Rheumatol..

[115] Adlan A., Paton J.F.R., Lip G.Y.H., Kitas G.D., Fisher J.P. (2016). Increased sympathetic nerve activity and reduced cardiac baroreflex sensitivity in rheumatoid arthritis. J. Physiol..

[116] van Maanen M.A., Vervoordeldonk M.J., Tak P.P. (2009). The cholinergic anti-inflammatory pathway: Towards innovative treatment of rheumatoid arthritis. Nat. Rev. Rheumatol..

[117] Van Maanen M.A., Stoof S.P., LaRosa G.J., Vervoordeldonk M.J., Tak P.P. (2010). Role of the cholinergic nervous system in rheumatoid arthritis: Aggravation of arthritis in nicotinic acetylcholine receptor 7 subunit gene knockout mice. Ann. Rheum. Dis..

[118] Lazzerini P.E., Acampa M., Capecchi P.L., Hammoud M., Maffei S., Bisogno S., Barreca C., Galeazzi M., Laghi-Pasini F. (2013). Association between high sensitivity C-reactive protein, heart rate variability and corrected QT interval in patients with chronic inflammatory arthritis. Eur. J. Intern. Med..

[119] Koopman F., Tang M., Vermeij J., de Hair M., Choi I., Vervoordeldonk M., Gerlag D., Karemaker J., Tak P. (2016). Autonomic Dysfunction Precedes Development of Rheumatoid Arthritis: A Prospective Cohort Study. eBioMedicine.

[120] Liu Y., Ho R.C.-M., Mak A. (2011). The role of interleukin (IL)-17 in anxiety and depression of patients with rheumatoid arthritis. Int. J. Rheum. Dis..

[121] Johnson R.L., Wilson C.G. (2018). A review of vagus nerve stimulation as a therapeutic intervention. J. Inflamm. Res..

[122] Rahman A., Isenberg D. (2008). Systemic Lupus Erythematosus. N. Engl. J. Med..

[123] Maule S., Quadri R., Mirante D., Pellerito R.A., Marucco E., Marinone C., Vergani D., Chiandussi L., Zanone M.M. (1997). Autonomic nervous dysfunction in systemic lupus erythematosus (SLE) and rheumatoid arthritis (RA): Possible pathogenic role of autoantibodies to autonomic nervous structures. Clin. Exp. Immunol..

[124] Stojanovich L., Milovanovich B., De Luka S., Popovich-Kuzmanovich D., Bisenich V., Djukanovich B., Randjelovich T., Krotin M. (2007). Cardiovascular autonomic dysfunction in systemic lupus, rheumatoid arthritis, primary Sjögren syndrome and other autoimmune diseases. Lupus.

[125] Capellino S., Lowin T., Angele P., Falk W., Grifka J., Straub R.H. (2007). Increased chromogranin A levels indicate sympathetic hyperactivity in patients with rheumatoid arthritis and systemic lupus erythematosus. J. Rheumatol..

[126] Laversuch C.J., Seo H., Modarres H., A Collins D., McKenna W.J., E Bourke B. (1997). Reduction in heart rate variability in patients with systemic lupus erythematosus. J. Rheumatol..

[127] Louthrenoo W., Ruttanaumpawan P., Aramrattana A., Sukitawut W. (1999). Cardiovascular autonomic nervous system dysfunction in patients with rheumatoid arthritis and systemic lupus erythematosus. QJM: Int. J. Med..

[128] Aydemir M., Yazisiz V., Basarici I., Avci A., Erbasan F., Belgi A., Terzioglu E. (2009). Cardiac autonomic profile in rheumatoid arthritis and systemic lupus erythematosus. Lupus.

[129] Poliwczak A., Waszczykowska E., Dziankowska-Bartkowiak B., Koziróg M., Dworniak K. (2017). The use of heart rate turbulence and heart rate variability in the assessment of autonomic regulation and circadian rhythm in patients with systemic lupus erythematosus without apparent heart disease. Lupus.

[130] Yorgun H., Canpolat U., Aytemir K., Ateş A.H., Kaya E.B., Akdoğan A., Sunman H., Canpolat A.G., Çalgüneri M., Kabakçı G. (2011). Evaluation of cardiac autonomic functions in patients with systemic lupus erythematosus. Lupus.

[131] Malliani A., Montano N. (2002). Emerging Excitatory Role of Cardiovascular Sympathetic Afferents in Pathophysiological Conditions. Hypertension.

[132] Carandina A., Rodrigues G.D., Di Francesco P., Filtz A., Bellocchi C., Furlan L., Carugo S., Montano N., Tobaldini E. (2021). Effects of transcutaneous auricular vagus nerve stimulation on cardiovascular autonomic control in health and disease. Auton. Neurosci..

[133] Thayer J.F., Yamamoto S.S., Brosschot J.F. (2010). The relationship of autonomic imbalance, heart rate variability and cardiovascular disease risk factors. Int. J. Cardiol..

[134] Restivo V., Candiloro S., Daidone M., Norrito R., Cataldi M., Minutolo G., Caracci F., Fasano S., Ciccia F., Casuccio A. (2021). Systematic review and meta-analysis of cardiovascular risk in rheumatological disease: Symptomatic and non-symptomatic events in rheumatoid arthritis and systemic lupus erythematosus. Autoimmun. Rev..

[135] Schoenfeld S.R., Kasturi S., Costenbader K.H. (2013). The epidemiology of atherosclerotic cardiovascular disease among patients with SLE: A systematic review. Semin. Arthritis Rheum..

[136] Pham G.S., Wang L.A., Mathis K.W. (2018). Pharmacological potentiation of the efferent vagus nerve attenuates blood pressure and renal injury in a murine model of systemic lupus erythematosus. Am. J. Physiol. Integr. Comp. Physiol..

[137] Fairley A.S., Mathis K.W. (2017). Cholinergic agonists reduce blood pressure in a mouse model of systemic lupus erythematosus. Physiol. Rep..

[138] Nomura A., Kishimoto M., Takahashi O., Deshpande G.A., Yamaguchi K., Okada M. (2013). Prolongation of heart rate-corrected QT interval is a predictor of cardiac autonomic dysfunction in patients with systemic lupus erythematosus. Rheumatol. Int..

[139] Sallam H., McNearney T.A., Chen J.D.Z. (2006). Systematic review: Pathophysiology and management of gastrointestinal dysmotility in systemic sclerosis (scleroderma). Aliment. Pharmacol. Ther..

[140] Herrick A.L. (2005). Pathogenesis of Raynaud’s phenomenon. Rheumatology.

[141] Di Franco M., Paradiso M., Riccieri V., Basili S., Mammarella A., Valesini G. (2007). Autonomic dysfunction and microvascular damage in systemic sclerosis. Clin. Rheumatol..

[142] Gigante A., Margiotta D.P.E., Navarini L., Liberatori M., Barbano B., Tubani L., Afeltra A., Rosato E. (2018). Parasympathetic activity increases with digital microvascular damage and vascular endothelial growth factor in systemic sclerosis. Clin. Exp. Rheumatol..

[143] Dessein P.H., Joffe B.I., Metz R.M., Millar D.L., Lawson M., Stanwix A.E. (1992). Autonomic dysfunction in systemic sclerosis: Sympathetic overactivity and instability. Am. J. Med..

[144] Othman K.M., Assaf N.Y., Farouk H.M., Hassan I.M.A. (2010). Autonomic Dysfunction Predicts Early Cardiac Affection in Patients with Systemic Sclerosis. Clin. Med. Insights Arthritis Musculoskelet. Disord..

[145] Cozzolino D., Naclerio C., Iengo R., D’Angelo S., Cuomo G., Valentini G. (2002). Cardiac autonomic dysfunction precedes the development of fibrosis in patients with systemic sclerosis. Rheumatology.

[146] Rodrigues G.D., Tobaldini E., Bellocchi C., Santaniello A., Caronni M., Severino A., Froldi M., Beretta L., Soares P.P.D.S., Montano N. (2019). Cardiac autonomic modulation at rest and during orthostatic stress among different systemic sclerosis subsets. Eur. J. Intern. Med..

[147] Gigante A., Galea N., Borrazzo C., Tubani L., Liberatori M., Ciolina F., Fiorelli A., Romaniello A., Barbano B., Romaggioli L. (2019). Role of autonomic dysfunction in the regulation of myocardial blood flow in systemic sclerosis evaluated by cardiac magnetic resonance. Int. J. Rheum. Dis..

[148] Zlatanovic M., Tadic M., Celic V., Ivanovic B., Stevanovic A., Damjanov N. (2016). Cardiac mechanics and heart rate variability in patients with systemic sclerosis: The association that we should not miss. Rheumatol. Int..

[149] Tadic M., Zlatanovic M., Cuspidi C., Stevanovic A., Celic V., Damjanov N., Kocijancic V. (2017). Systemic sclerosis impacts right heart and cardiac autonomic nervous system. J. Clin. Ultrasound.

[150] Tadic M., Zlatanovic M., Cuspidi C., Ivanovic B., Stevanovic A., Damjanov N., Kocijancic V., Celic V. (2017). The relationship between left ventricular deformation and heart rate variability in patients with systemic sclerosis: Two- and three-dimensional strain analysis. Int. J. Cardiol..

[151] Ciftci O., Onat A.M., Yavuz B., Akdogan A., Aytemir K., Tokgozoglu L., Sahiner L., Deniz A., Ureten K., Kizilca G. (2007). Cardiac repolarization abnormalities and increased sympathetic activity in scleroderma. J. Natl. Med. Assoc..

[152] Pancera P., Sansone S., Presciuttini B., Montagna L., Cerù S., Lunardi C., Lechi A. (1999). Autonomic nervous system dysfunction in sclerodermic and primary Raynaud’s phenomenon. Clin. Sci..

[153] Lock G., Straub R.H., Zeuner M., Antoniou E., Holstege A., Schölmerich J., Lang B. (1998). Association of autonomic nervous dysfunction and esophageal dysmotility in systemic sclerosis. J. Rheumatol..

[154] DiRenzo D., Russell J., Bingham C.O., McMahan Z. (2019). The Relationship between Autonomic Dysfunction of the Gastrointestinal Tract and Emotional Distress in Patients with Systemic Sclerosis. JCR J. Clin. Rheumatol..

[155] Adler B., Russell J.W., Hummers L.K., Mcmahan Z.H. (2018). Symptoms of Autonomic Dysfunction in Systemic Sclerosis Assessed by the COMPASS-31 Questionnaire. J. Rheumatol..

[156] Cerinic M.M., Generini S., Pignone A., Casale R. (1996). THE NERVOUS SYSTEM IN SYSTEMIC SCLEROSIS (SCLERODERMA): Clinical Features and Pathogenetic Mechanisms. Rheum. Dis. Clin. N. Am..

[157] Carandina A., Bellocchi C., Rodrigues G.D., Beretta L., Montano N., Tobaldini E. (2021). Cardiovascular Autonomic Control, Sleep and Health Related Quality of Life in Systemic Sclerosis. Int. J. Environ. Res. Public Health.

[158] Lee S.-P., Kim S.-H., Kim T.H., Sohn J.W., Shin D.H., Park S.S., Yoon H.J. (2010). A Case of Mexiletine-induced Hypersensitivity Syndrome Presenting as Eosinophilic Pneumonia. J. Korean Med. Sci..

[159] Kees M.G., Pongratz G., Kees F., Schölmerich J., Straub R.H. (2003). Via β-adrenoceptors, stimulation of extrasplenic sympathetic nerve fibers inhibits lipopolysaccharide-induced TNF secretion in perfused rat spleen. J. Neuroimmunol..

[160] Ghia J.-E., Blennerhassett P., Collins S.M. (2007). Vagus nerve integrity and experimental colitis. Am. J. Physiol. Liver Physiol..

[161] Koopman F.A., Chavan S.S., Miljko S., Grazio S., Sokolovic S., Schuurman P.R., Mehta A.D., Levine Y., Faltys M., Zitnik R. (2016). Vagus nerve stimulation inhibits cytokine production and attenuates disease severity in rheumatoid arthritis. Proc. Natl. Acad. Sci. USA.

[162] Vagus Nerve Stimulation in Rheumatoid Arthritis. https://clinicaltrials.gov/ct2/show/NCT00859859.

[163] Long Term Observational Study of the Safety and Efficacy of an Active Implantable Vagal Nerve Stimulation Device in Patients with Rheumatoid Arthritis. https://clinicaltrials.gov/ct2/history/NCT01552538?V_1=View.

[164] Safety and Efficacy Vagal Nerve Stimulation in Patients with Rheumatoid Arthritis. https://clinicaltrials.gov/ct2/show/NCT01552941.

[165] Vagus Nerve Stimulation a New Approach in the Treatment of Crohn’s Disease (VNS). https://clinicaltrials.gov/ct2/show/NCT01569503.

[166] Chakravarthy K., Chaudhry H., Williams K.A., Christo P.J. (2015). Review of the Uses of Vagal Nerve Stimulation in Chronic Pain Management. Curr. Pain Headache Rep..

[167] Napadow V., Edwards R.R., Cahalan C.M., Mensing G., Greenbaum S., Valovska A., Li A., Kim J., Maeda Y., Park K. (2012). Evoked Pain Analgesia in Chronic Pelvic Pain Patients Using Respiratory-Gated Auricular Vagal Afferent Nerve Stimulation. Pain Med..

[168] Janner H., Klausenitz C., Gürtler N., Hahnenkamp K., Usichenko T.I. (2018). Effects of Electrical Transcutaneous Vagus Nerve Stimulation on the Perceived Intensity of Repetitive Painful Heat Stimuli. Anesthesia Analg..

[169] Franzini A., Messina G., Marras C., Savino M., Miniati M., Bugiani O., Broggi G. (2008). Hamilton Rating Scale for Depression-21 Modifications in Patients With Vagal Nerve Stimulation for Treatment of Treatment-Resistant Depression: Series Report. Neuromodulation Technol. Neural Interface.

[170] Frangos E., Ellrich J., Komisaruk B.R. (2014). Non-invasive Access to the Vagus Nerve Central Projections via Electrical Stimulation of the External Ear: fMRI Evidence in Humans. Brain Stimul..

[171] Ramkissoon C.M., Güemes A., Vehi J. (2021). Overview of therapeutic applications of non-invasive vagus nerve stimulation: A motivation for novel treatments for systemic lupus erythematosus. Bioelectron. Med..

[172] Aranow C., Atish-Fregoso Y., Lesser M., Mackay M., Anderson E., Chavan S., Zanos T.P., Datta-Chaudhuri T., Bouton C., Tracey K.J. (2020). Transcutaneous auricular vagus nerve stimulation reduces pain and fatigue in patients with systemic lupus erythematosus: A randomised, double-blind, sham-controlled pilot trial. Ann. Rheum. Dis..

[173] Sallam H., McNearney T.A., Doshi D., Chen J.D.Z. (2007). Transcutaneous Electrical Nerve Stimulation (TENS) Improves Upper GI Symptoms and Balances the Sympathovagal Activity in Scleroderma Patients. Am. J. Dig. Dis..

